# Retraction of: Glypican-1-targeted and gemcitabine-loaded liposomes enhance tumor-suppressing effect on pancreatic cancer

**DOI:** 10.18632/aging.206242

**Published:** 2025-04-30

**Authors:** Yu Mu, Dezhi Wang, Liangyu Bie, Suxia Luo, Xiaoqian Mu, Yanqiu Zhao

**Affiliations:** 1 Department of Oncology, Affiliated Cancer Hospital of Zhengzhou University, Henan Cancer Hospital, Zhengzhou, Henan Province, China; 2 East China Normal University, Shanghai, China

**Keywords:** liposome, phosphatidylinositol proteosan-1, gemcitabine, orthotopic pancreatic cancer mice, pancreatic cancer

**This article has been retracted:** Aging has completed its investigation of this paper. Several concerns were raised about this paper, including overlap with unrelated papers from different institutions. We found that a few tumor images in Figure 5B “GPC1-LP (GEM) inhibited tumor growth of the orthotopic pancreatic cancer mice” are duplications of tumor images in Figure 6A published earlier in an unrelated paper [1] and Figures 6B, 6b, 6B and 4G published in four more unrelated papers [2–5], some of which already have been retracted. In addition, Figure 1B “Cumulative drug release profiling of LP (GEM) and GPC1-LP (GEM) in phosphate-buffer saline (PBS, pH 7.4 and pH 5.0)” contains the same graph as unrelated paper [6]. The Scientific Integrity office at Aging contacted the authors and their institution. The authors confirmed that tumor images were inappropriately used and agreed with the Editorial decision to retract this paper. The Scientific Integrity office notified the authors’ Institutions about this retraction and added their names to an Editorial Warning list.
